# Subcortical orientation biases explain orientation selectivity of visual cortical cells

**DOI:** 10.14814/phy2.12374

**Published:** 2015-04-08

**Authors:** Trichur R Vidyasagar, Jaikishan Jayakumar, Errol Lloyd, Ekaterina V Levichkina

**Affiliations:** 1 Department of Optometry & Vision Sciences, University of MelbourneParkville, Victoria, Australia; 2 Melbourne Neuroscience Institute, University of MelbourneParkville, Victoria, Australia; 3 Institute for Information Transmission Problems (Kharkevich Institute), Russian Academy of SciencesMoscow, Russia

**Keywords:** Lateral geniculate nucleus, kainic acid, muscimol, optical imaging, orientation selectivity, primary visual cortex

## Abstract

The primary visual cortex of carnivores and primates shows an orderly progression of domains of neurons that are selective to a particular orientation of visual stimuli such as bars and gratings. We recorded from single-thalamic afferent fibers that terminate in these domains to address the issue whether the orientation sensitivity of these fibers could form the basis of the remarkable orientation selectivity exhibited by most cortical cells. We first performed optical imaging of intrinsic signals to obtain a map of orientation domains on the dorsal aspect of the anaesthetized cat's area 17. After confirming using electrophysiological recordings the orientation preferences of single neurons within one or two domains in each animal, we pharmacologically silenced the cortex to leave only the afferent terminals active. The inactivation of cortical neurons was achieved by the superfusion of either kainic acid or muscimol. Responses of single geniculate afferents were then recorded by the use of high impedance electrodes. We found that the orientation preferences of the afferents matched closely with those of the cells in the orientation domains that they terminated in (Pearson's *r* = 0.633, *n* = 22, *P* = 0.002). This suggests a possible subcortical origin for cortical orientation selectivity.

## Introduction

**Figure 1:**
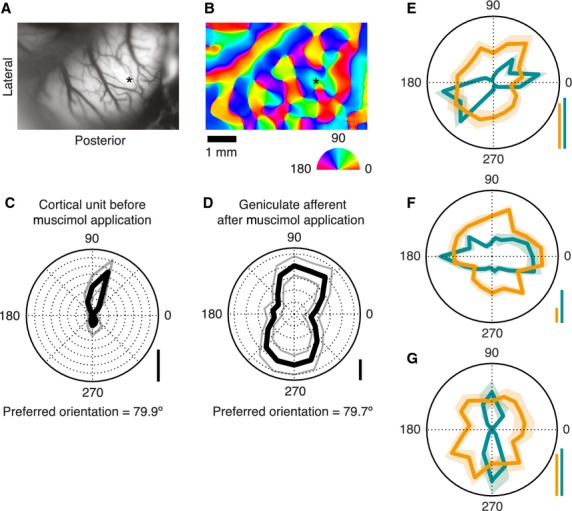
Orientation sensitivity of cat area 17 cells and of the geniculate afferents in the near vicinity. (A) Surface image of area 17. (B) Orientation domain map of the cortex color coded according to the semicircular color wheel below. The asterisks in A and B mark the site of a perpendicular electrode penetration. (C) Polar plot of a cortical cell's response to stimuli of different orientations, recorded from the penetration marked in A and B. Gray trace marks ± SEM. (D) As with C, but for an afferent fiber, isolated after application of Muscimol. (E–F) Overlaid polar diagrams of responses of cortical-afferent pairs, each pair recorded from a single penetration. Green traces refer to cortical units, orange traces to afferent units, with ± SEM. For C–G, real spike rates are denoted at the lower right of each plot by vertical scale bars indicating length of each data point from the center of the plot and representing 40 spikes/s. For E–G, scale bars are colored as per their corresponding unit.

Cells in the primary visual (striate) cortex of mammals show a remarkable degree of selectivity to the orientation of a visual stimulus such as a line or an edge (Hubel and Wiesel 1962). Debate has raged for decades whether this selectivity critically depends upon intracortical, especially inhibitory, mechanisms, the pattern of termination of excitatory afferents from the lateral geniculate nucleus (LGN) in the visual thalamus, the mild orientation sensitivity already exhibited by retinal and geniculate neurons or a combination of these (Hubel and Wiesel 1962; Creutzfeldt et al. 1974; Sillito et al. 1980; Ferster et al. 1996; Vidyasagar et al. 1996; Crook et al. 1998; Priebe and Ferster 2012). Some studies had reported the presence of an orientation-tuned feedforward excitatory response in cortical cells when intracortical activity was suppressed (Ferster et al. 1996; Li et al. 2013; Lien and Scanziani 2013) claiming either an excitatory convergence from a number of LGN cells with receptive fields along a row in visual space (Hubel and Wiesel 1962), or excitatory projections from LGN afferents with spatially offset ON and OFF centers (Jin et al. 2011; Paik and Ringach 2011, 2012; Wang et al. 2015) as the basis for the generation of the orientation selectivity of cortical cells. However, these interpretations had overlooked the possibility that intracortical inhibition that always accompanies excitatory signals from natural visual stimulation, given the mild degrees of orientation selectivity that single geniculate cells exhibit (Vidyasagar and Urbas 1982; Shou and Leventhal 1989), could lead to the emergence of sharp orientation selectivity in the cortical cell's excitatory response (Vidyasagar 1987; Thompson et al. 1994; Kuhlmann and Vidyasagar 2011; Viswanathan et al. 2011). One consequence of this scheme would be a close match between the preferred stimulus orientation of a geniculostriate afferent and that of the cortical cells that it projects to. No such relationship would be predicted by a scheme that depends either on an excitatory convergence from LGN cells (Hubel and Wiesel 1962) or on spatially offset ON and OFF inputs (Jin et al. 2011; Paik and Ringach 2011, 2012; Wang et al. 2015). To test the role of such subcortical orientation biases, we recorded from single afferents in the geniculate input layer 4 after pharmacological silencing of cortical cells, using kainic acid or muscimol (see Methods and Fig.1). For each penetration, the preferred stimulus orientation of geniculate afferents was compared to the preferred stimulus orientation of cells in the same site established earlier by either optical imaging of intrinsic signals or single cell recording using microelectrodes or both.

## Methods

### Surgical preparation

Experiments were performed on six domestic cats (Felix catus) and all procedures were approved by the University of Melbourne Animal Experimentation Ethics Committee and conformed to the animal ethics guidelines of the National Health and Medical Research Council of Australia. Following initial intramuscular administration of Ketamine (15 mg/kg, Ketamil, Parnell laboratories Pty Ltd, Alexandria, NSW, Australia) and Xylazine (2 mg/kg, Illium Xylazil, Bayer, Pymble, NSW, Australia), each animal underwent a tracheostomy and cephalic venous cannulation to deliver drugs and nutrients. The animal was artificially ventilated and the anesthesia was maintained with Isofluorane (0.5–2%) in a mixture of nitrous oxide and oxygen (70:30%). Skeletal muscle paralysis was induced and maintained with Vecuronium bromide (0.2 mg/kg/h; Norcuron, Organon, Lane Cove, NSW, Australia). The end-tidal carbon dioxide was kept at 3.4–3.8% and the sub-scapular temperature was maintained at 36°C using a servo-controlled thermoelectric blanket. The electroencephalogram and the electrocardiogram were used to adjust the delivery of anesthetic gases to maintain an adequate level of anesthesia.

After retracting the nictitating membrane, using phenylephrine hydrochloride (Neosynephrine, Sanofi, Whinthorp, Macquairie Park, NSW, Australia), the eyes were dilated using Atropine (Atropot 0.5%, Sigma-Aldritch, CastleHill, NSW, Australia). The eyes were refracted using streak retinoscopy and rigid gas permeable contact lenses (Capriconia Lenses, Brisbane, QLD, Australia) and appropriate optical lenses were used to protect the eyes and correct for refractive errors, respectively. A craniotomy was performed between Horsley–Clarke coordinates AP −8 and +2 and LM 0.5 and 6 mm from the midline to access the dorsal aspect of area 17. A metal chamber was mounted around this opening using dental cement (Vertex, Dentimex BV, Zeist, the Netherlands) and filled with silicone oil (Poly dimethyl siloxane 200, Sigma-Aldrich, Australia) following a durotomy and finally sealed tight with a cover glass to enable optical imaging. After optical imaging, the cover glass was removed for microelectrode penetrations and the cortex was covered with 2% agar during the electrophysiological recordings.

### Optical imaging

The orientation domains of the exposed area 17 were mapped using optical imaging of intrinsic signals, which were obtained by means of an Imager 2001 (Optical Imaging, Rehevot, Israel) following standard procedures (Buzas et al. 1998). Briefly, cortical surface details were first mapped using 545 nm (green) light and a CCD camera (Pulnix, Santa Clara, CA) with tandem optics (SMC pentax, 1:1.2, *f* = 50 mm). This image (Fig.1A) was used to align the optical images with the surface landmarks. Intrinsic signals were acquired using 630 nm light and the camera focused 600 microns below the cortical surface in response to high contrast, full-field, square wave visual gratings (0.2–1.0 cycles/deg moving at 1.5 Hz). The stimuli were generated on a Barco monitor (80 Hz Frame rate, Barco Video and Communications, Kortrijk, Belgium) by a visual stimulus generator (Visage, Cambridge Research systems, Rochester, UK). Responses to eight different orientation-directions, separated by 22.5 degrees, were each obtained for a presentation time of 7.3 sec followed by an interstimulus interval of 10 sec, during which the animal viewed a blank screen of the same average luminance. The optical images obtained were digitized and single condition maps (sum of all images obtained for the same orientation) and a “cocktail” (sum of all images) were used to compute orientation maps, where orientations of the pixels ranged from 0° to 179°. An example of the final orientation domain map with the orientation key for the pseudocolor code is given in Figure1B. The orientation domains and pinwheel centers marked on these maps were overlaid on the green image to mark the positions with reference to surface blood vessels for targeting microelectrode penetrations.

### Electrophysiological recordings

Single unit electrophysiological recordings were carried out using high impedance lacquer-coated tungsten electrodes (12–18 MOhms, FHC Inc, Bowdoin, ME). The high impedance of the electrodes was critical in our experiment to ensure that we were able to record and isolate “single” geniculate afferents. In a previous study by Chapman et al. (1991) using a similar protocol (i.e., kainic acid or muscimol to silence the cortex), the electrode impedances were 1–2 MOhms, which would have led to recordings of mostly multiple afferents. We first roughly identified area 17 by its known topography (medial and posterior within our craniotomy) and by its preference for higher spatial frequency and slower angular velocity of moving visual stimuli in comparison to the more antero-laterally located area 18. The locations of electrode penetrations were later confirmed by histology. Visual stimuli used in the recordings were generated using a visual stimulus generator (Visage). Single-unit activities were amplified using an AM Systems amplifier (Model 1800, AM Systems, Sequim, WA) and recorded using a CED 1401 data acquisition system (Cambridge Electronic Design Ltd, Cambridge, UK).

Extracellular electrophysiological recordings were obtained from electrode penetrations made in large orientation preference domains as discerned from optical imaging (see above). In 10 cases, in order to confirm the orientation preference determined from optical imaging and to provide a more accurate estimate of the domain's preference, recordings were made from single units. Lacquer-coated tungsten electrodes were inserted perpendicularly to the cortical surface at locations recorded on the aforementioned reference image according to vascular landmarks. Following isolation of the unit and manual receptive field plotting from an audio feed of the amplifier's output, responses to sweeping bars of different orientations were recorded. Stimuli were presented on the Barco monitor, generated from a PC with a Vistaboard (Truevision, Ottawa, Ontario, Canada) for approximately half the data and the same equipment (Visage) as for optical imaging for the remainder. The bars were presented at 80–100% contrast against a gray background luminance of 17.4 cd·m^−2^ at a distance of 1 meter. The bars were darker or lighter than background, depending on which elicited the greater response, with 10° length, 0.25 to 1° width and sweeping at 2–4° per s across a 10° arc. The bar's sweeps were biphasic (i.e. back and forth, once each), and presented at 9 orientations between 0° and 180°, separated by 20° steps. Each orientation was presented 10 times.

### Pharmacological silencing of area 17

Following optical imaging and in some cases, electrophysiological recordings to measure the orientation selectivity of striate cortical cells, we silenced the activity of the exposed dorsal aspect of area 17 either by using kainic acid or muscimol. Kainic acid was applied in four experiments and muscimol was used for inactivation in two animals. Kainic acid solution (10 mmol/L in 0.9% saline) was superfused on the exposed cortex for 12 h at the rate of 74 *μ*L/h, as per methods used in a similar study (Chapman et al., 1991). Kainic acid is a glutamate agonist, which can be used as an excitatory neurotoxin to kill neurons. It acts by activating the kainite receptors that are present on soma, but not on axons and thus spares the afferent terminals that terminate in the cortex. In two experiments, we used Muscimol, a long acting GABA agonist, which silences the responses of cortical neurons, yet maintains the integrity of the neruopile structure. A quantity of 74 *μ*L of 50 mmol/L Muscimol in 0.9% sterile saline solution was superfused onto the cortex at the penetration site of the electrode, twice, an hour apart. Recording commenced 2 h after the first application. Receptive fields of the recorded cortical cells and the afferents were plotted onto a tangent screen. They showed considerable overlap well within the receptive field scatter expected for the recorded eccentricities in the cat area 17 (Albus 1975). Electrolytic lesions (6 *μ*A for 6 sec) were made at the sites of recordings from the afferents and later histological reconstructions identified these sites as being limited to layer IV. Though identification was somewhat difficult with the kainic acid experiments due to the loss of most cell bodies, extrapolation from adjacent unaffected cortical laminae, combined with estimates of the depth of electrode tips both from the lesions and the micrometer readings led to confirmation of the laminar locations of the recorded afferents.

## Results

**Figure 2:**
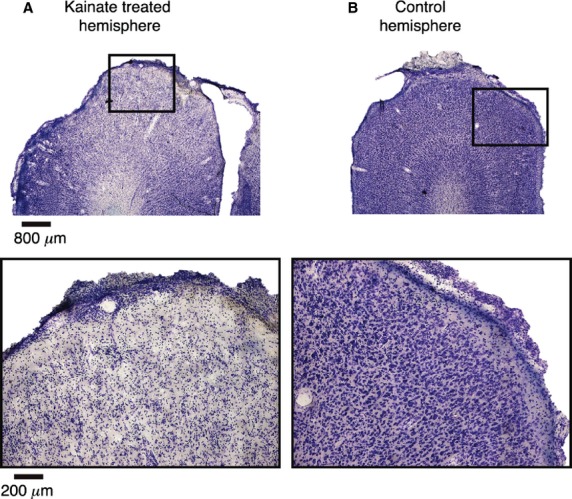
Effect of kainic acid on the cortex. (A) Single 50 *μ*m thick slice of area 17, stained for Nissl substance, using classical histological techniques, following topical application of kainic acid to the cortical surface in vivo. Top image is at lower magnification and demonstrates the excitotoxic ablation of the affected area as indicated by the decrease in the density of the Nissl stain in the superior portion relative to the lateral and medial extents of the cortex. The bottom image is of the region of the top image enclosed by the black box, which was most affected, viewed under higher magnification to further demonstrate the sparseness of Nissl bodies. (B) As with A, but image of the contralateral cortex of the same animal, on which no kainic acid was applied. The density of Nissl bodies relative to A indicates the extent and efficacy of the ablation.

After identifying the orientation preferences of the cortical domain, we used kainic acid (four animals) or muscimol solution (two animals) to silence the responses of cortical neurons. Figure2 shows an example of the extensive damage caused by kainic acid. Microscopic examination of the surface of the dorsal operculum that was exposed had only a sparse distribution of neurons that survived the kainate application.

**Figure 3:**
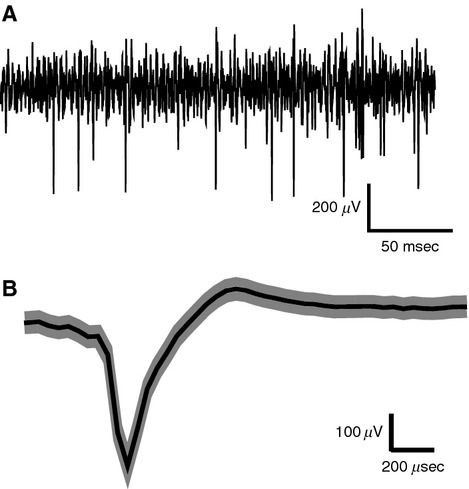
Isolation of spikes from afferent fibers. (A) Example of the response of a single afferent with action potentials recorded with a bandpass filter (300–3000 Hz) and digitally sampled every 75 *μ*s. (B) Average shape of the spikes recorded from a minimally filtered version of the recording channel shown in A (1–5000 Hz) with shaded regions marking ± SEM. Negative is shown upwards in both A and B. The spike's quick rising phase of maximum 150 *μ*s, short duration of maximum 300 *μ*s, the monophasic nature, the high signal to noise ratio and the consistent shape and amplitude indicate that the spikes are most likely from a single isolated fiber. Please note that due to the digitization at 13.3 KHz, changes in the potential faster than 75 *μ*s is not captured by the trace.

**Figure 4:**
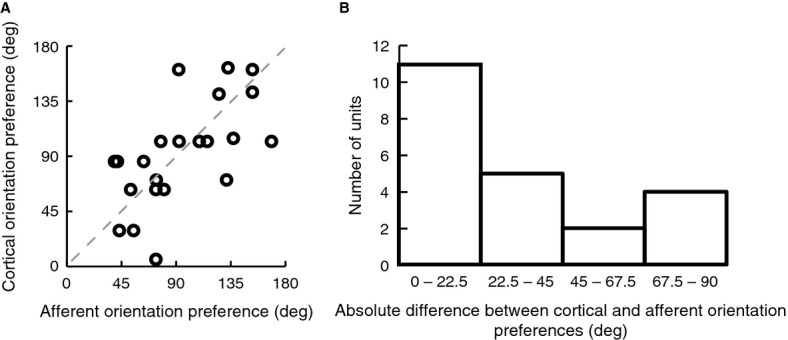
Correlation between orientation preferences of geniculate afferents and the orientation preference of their target cortical domains. (A) Scatter plot of the preferred cortical and afferent orientation for all pairs, demonstrating a significant correlation (Pearson's *r* = 0.633, *n* = 22, *P* = 0.002). The dotted line represents the identity line. (B) Distribution of absolute differences between the preferred orientations of afferents and their cortical targets, demonstrating a significant tendency toward zero when tested against a random distribution.

After pharmacological silencing, we recorded using high impedance electrodes the activity of single geniculate afferents that were usually still responsive within the exposed area 17. Figure3A shows a recording from a putative afferent obtained after pharmacological silencing. Geniculate afferents were identified by their response characteristics, in particular, their monocularity, broad or no orientation tuning, higher spontaneous discharge, and relatively faster rising phase of the spike and shorter duration of the action potentials in comparison to soma spikes (Bishop et al. 1962). Figure3B shows the mean waveform with standard error of an afferent recorded after kainate application. It was important that we recorded from single isolated afferents and not from multiple afferents, since an orientation bias of a multiunit response cannot distinguish between the presence of similar orientation biases in all the fibers recorded or due to elongated scatter of essentially circular afferent fields. Our results show that the orientation preferences of single geniculate afferents match closely with those of the cells in the cortical domain that they terminate in. Panels C and D of Figure1 constitute an example pair of units, where the orientation preference of an afferent unit matches closely the orientation preference of a cortical unit recorded from the same track. Panels E-G in Figure1 show three other pairs of examples from three different animals, of an LGN afferent (orange) and a cortical cell (green) that were recorded within the same track. Each of them shows that the orientation preference of the afferent unit matches closely with that of the cortical unit. The pooled data (Fig.4A) show a significant relationship between the orientation preferences of single geniculate afferents and the orientation preferences of the orientation domain that they terminate in (Pearson's *r* = 0.633, *n* = 22, *P* = 0.002). Eleven out of the 22 afferent units showed an orientation preference which was <22.5 degrees from the optimum orientation of the cortical domain, a result which is significantly different from chance (Chi square = 8.182, *P* = 0.041; see Fig.4B).

## Discussion

Our results address a central issue with regard to how thalamic inputs constrain and determine the responses of cells in the sensory cortices. The model originally proposed by Hubel and Wiesel to explain the orientation selectivity of cells in the primary visual cortex of cats (Hubel and Wiesel 1962) and macaques (Hubel and Wiesel 1968) relies purely on the excitatory convergence of geniculate afferents which themselves are assumed to be not orientation selective and having only circular receptive fields. However, over the last 30 years, much evidence has accumulated for the presence of mild degrees of orientation selectivity in the retina (Levick and Thibos 1980; Passaglia et al. 2002) and LGN of every species studied so far (Vidyasagar and Urbas 1982; Shou and Leventhal 1989; Smith et al. 1990; Thompson et al. 1994; Scholl et al. 2013; Van Hooser et al. 2013; : Zhao et al. 2013). In both the retina (Levick and Thibos 1980) and the LGN (Vidyasagar and Heide 1984) of the cat, it has been shown that the orientation selectivity increases considerably with higher spatial frequency stimuli. Furthermore, the nonspecific or broadly tuned inhibition that all layer four striate cells receive via fast-spiking interneurons (Pei et al. 1994; Mariño et al. 2005; Cardin et al. 2007; Nowak et al. 2008) suppresses the responses to low spatial frequency stimuli (Vidyasagar and Mueller 1994) and leaves the cortical cell tuned to both spatial frequency and orientation. This has been demonstrated also in a recent computational study (Kuhlmann and Vidyasagar 2011). This scheme, in contrast to the classical Hubel and Wiesel model, predicts a clear relationship between the preferred stimulus orientation of a geniculate afferent and the cortical cell that it projects to. This prediction is borne out by our finding of both geniculate afferents and cortical cells at a striate cortical site preferring in most cases the same stimulus orientation, whereas the excitatory convergence model makes no such prediction.

There is, however, one conundrum posed by a scheme that relies on the orientation selectivity of single retinal cells to elaborate the whole variety of preferred orientations that are represented for any one visual field locus on the cortex. This issue is addressed by the argument that orientation information, if already coded at the level of the retina, can indeed be coded only by a limited number of rather broadly tuned channels, with the full gamut of orientations being elaborated in the cortex from these primary orientations (Vidyasagar 1985, 1987; Vidyasagar et al. 1996; Kuhlmann and Vidyasagar 2011). In this respect, coding orientation is analogous to the coding of color, where three broadly tuned cone photoreceptors capture the entire visible spectrum for later elaboration of all the myriad hues in the cortex. The retinal detectors, be they for color or for orientation, cannot be sharply tuned, if the system has to preserve both sensitivity as well as resolution. This framework is consistent with the preference for just a few orientations exhibited by many of the cells in the retina (Levick and Thibos 1980; Leventhal and Schall 1983; Schall et al. 1986) and LGN (Vidyasagar and Urbas 1982; Shou and Leventhal 1989; Smith et al. 1990) that are broadly tuned for orientation. That preferences for nonradial orientations are generated by inputs from the radially biased afferents and the afferents tuned to other, possibly orthogonal (Shou and Leventhal 1989), orientations, is reflected in the departures from the identity line for some of the data points in Figure4A.

An earlier study in ferrets using a similar protocol (Chapman et al., 1991) of inactivating cell responses and leaving fiber responses intact had shown that the locations of afferent receptive fields in a vertical penetration seem to be elongated often in the same axis as the preferred orientation of the cell in the same penetration and was interpreted as supporting evidence to the Hubel and Wiesel model. This result seemed to be consistent with a subsequent cross-correlation study of simultaneous recordings from the LGN and area 17 in the cat (Reid and Alonso 1995). However, not only do intracellular recordings reveal only a slight elongation of the excitatory sub-region (Creutzfeldt et al. 1974; Pei et al. 1994), but a more recent study using spike-triggered averaging of striate cortical local field potentials generated from thalamic neurons (Jin et al. 2011) has also found the scatter to be much less than would be required by the excitatory convergence model. It is to be noted that in both these studies (Chapman et al. 1991; Jin et al. 2011), cortical responses were recorded with low impedance electrodes that could not have recorded single afferents and thus the match we found between the preferred stimulus orientations of single geniculate afferents and their putative targets would have been missed. It is also to be noted that since there is a dominance of the preference for one, namely radial, orientation in the afferents at any one visual field location, not only would the multiunit response from the afferents at a cortical site exhibit an orientation bias, but the uncertainties in estimation of receptive field centres in Chapman et al's study (1991) is also likely to be elongated in the radial axis. It is possible that these factors might have led Chapman et al. (1991) to the alternative conclusion that the orientation selectivity is generated by an excitatory convergence as proposed by Hubel and Wiesel (1962).

The possible retinogeniculate basis for cortical orientation selectivity suggested by our study is also reflected in the distribution of preferred orientations of cortical cells matching the retinal pattern (Leventhal 1983; Schall et al. 1986). Human psychophysical (Rovamo et al. 1982; Temme et al. 1985; Fahle 1986; Bennet and Banks 1991; Berardi and Fiorentini 1991; McGraw and Whitaker 1999; Westheimer 2003) and human and macaque imaging (Sasaki et al. 2006) studies also show a radial bias, especially away from the fovea, and in the fovea itself, most studies have reported an additional bias for just two orientations, namely the vertical and horizontal.

The close correspondence we find between the preferred orientation of LGN afferents and the nearby cortical cells lends credence to the idea that cortical orientation selectivity may be critically dependent on the orientation biases of LGN cells, to the extent that a pharmacologically induced reduction of orientation biases in the LGN could even reduce striate cortical orientation selectivity (Vidyasagar 1992).

## Conflict of Interest

None declared.
